# Spatial Distribution Characteristics and Influencing Factors of Traditional Villages in China

**DOI:** 10.3390/ijerph19084627

**Published:** 2022-04-12

**Authors:** Jiaojiao Bian, Wanxu Chen, Jie Zeng

**Affiliations:** Department of Geography, School of Geography and Information Engineering, China University of Geosciences, Wuhan 430078, China; bianjiaojiao1215@cug.edu.cn

**Keywords:** traditional villages, spatial distribution characteristics, influencing factors, spatial analysis, China

## Abstract

Traditional villages carry the essence of traditional culture, which is necessary for rural revitalisation. However, continuous urban expansion has resulted in the rapid decline and even disappearance of these villages in recent decades. It is necessary to analyse the spatial pattern and influencing factors for the protection and development of traditional villages. Previous studies focused on the value and theoretical protection mechanism of traditional villages in China, disregarding their spatial distribution characteristics and influencing factors. Thus, we employed a Geographic Information System and spatial analysis with mathematical statistics to analyse the characteristics of these villages. Moreover, we analysed the associated influencing factors both qualitatively and quantitatively. The results show that traditional villages were mainly distributed in the southeast of the Hu Line in China, with an unbalanced spatial distribution pattern and an agglomeration distribution tendency. In general, four major agglomeration areas of traditional villages formed at the junction of Hebei, Shandong, and Henan provinces; the border between the Guizhou, Guangxi, and Hunan provinces; the border between the Anhui, Zhejiang, and Jiangxi provinces; and northwestern and southeastern Yunnan provinces. Traditional villages also existed in areas with relief lower than 300 m, altitudes of less than 1000 m, and slopes of less than 10°. They were mostly distributed in subtropical and temperate zones. A positive correlation was found between traditional villages and the level of economic development, population, and human history; conversely, the transportation network was negatively correlated. This study reveals the complex and diverse characteristics of traditional villages and provides scientific suggestions for their future protection, development, and utilisation.

## 1. Introduction

Villages with material and intangible cultural heritage enriched with historical, cultural, scientific, artistic, and socioeconomic values are termed traditional villages [1]. They reflect the traditional style and national characteristics of their corresponding historical periods, typical farming civilisation, and traditional culture [2,3,4]. The development and protection of traditional villages are related to not only the survival of their traditional cultural diversity but also their successful reconstruction. Moreover, traditional villages can awaken the sense of a community of common destiny that traditional village culture and human culture are interconnected, and make new contributions to the cause of people’s exchanges and cultural interaction in regions along the Belt and Road Initiative [5]. However, due to rapid industrialisation and urbanisation in recent years, the decline and even disappearance of traditional villages have intensified. Existing studies on traditional villages lack a comprehensive analysis of the spatial distribution characteristics and influencing factors of traditional villages on a national scale. This study, therefore, used Geographic Information System (GIS) and spatial analysis with mathematical statistic methods to analyse the spatial distribution characteristics and influencing factors of traditional villages in China to provide theoretical support for their protection and development.

In China, traditional villages were formerly known as ‘ancient villages’, as they were formed and perfected in the ancient period. To date, a relatively complete village layout and structure have been preserved. China’s Traditional Village Protection and Development Research Centre officially renamed ‘ancient’ villages as ‘traditional villages’ in 2012. In late 2012, the first national list of traditional villages was released, indicating that efforts to protect traditional villages have been given legal status. By the end of 2019, there were five lists including a total of 6819 villages designated as national traditional villages. Traditional villages with historical and cultural significance in China have received increasing attention in the past years. Two key themes have been protection and usage [6]. Previous studies on traditional villages have made great progress in protecting their authenticity [2,7,8] and illuminating their spatial distribution characteristics [9,10,11,12], value identification and connotations to development [13,14,15], and tourism planning and development [6,16,17]. It is still necessary to conduct systematic research on the role of environmental factors and human historical factors in the protection of traditional villages in China. However, as only a few studies have examined the spatial distribution of traditional villages and their influencing factors at the regional scale [18,19], the topic needs further exploration.

In addition, previous studies have concluded that economic location, human history, market development, and physical geography are all closely related to the overall spatial distribution pattern of traditional villages [7,12,20,21,22]. Theoretically, the level of economic development should not pose an obstacle to the preservation of these villages. Therefore, traditional villages can be clustered in regions with a high level of economic development as well as economically underdeveloped regions [23]. A dense transportation network has also been proven to be negatively correlated with the preservation of traditional villages [24]. The preservation of historical and cultural values is an important factor affecting the preservation of traditional villages. For example, a large number of traditional villages emerged due to historical influences in the Taihu Basin and Miao customs in Guizhou province, respectively [25]. Previous studies have also found that traditional villages tend to be distributed in areas with a low population density, rather than near large cities or urban agglomerations with a dense population [7,26]. Natural elements have also played an important role in the distribution of traditional villages. Prior studies suggest that most traditional villages are located in areas with a low altitude and slope [27,28] as well as on sunny slopes and in areas with a topographic relief ranging from 100 m to 300 m [14]. It can be seen that socioeconomic factors, cultural and historical factors, and physical factors are all necessary to be used to analyse the distribution mechanism of traditional villages on a national scale.

Therefore, it is necessary to analyse the spatial pattern and influencing factors of traditional villages for their protection and development in the future. Specifically, the study on the spatial distribution of traditional villages can provide scientific reference for the future spatial planning and management of traditional villages. The discussion on the influencing factors of traditional villages can help the excavation of more traditional villages and the development of traditional villages. Protection of traditional villages not only relates to the conservation of Chinese traditional cultural diversity but also contributes to the reconstruction of rural areas in the world. Especially, it can make great contributions to deepening the exchanges and mutual learning of different civilizations in the countries and regions along the Belt and Road Regions.

This study has three main objectives: (1) analysing the spatial distribution characteristics of traditional villages in China from 2012 to 2019 using a series of GIS spatial analysis tools; (2) identifying the influencing factors of traditional villages in China from 2012 to 2019 using mathematical statistics methods; and (3) providing policies related to the development, utilisation, and protection of traditional villages in China.

## 2. Materials and Methods

### 2.1. Data Sources and Processing

**Figure 1:**
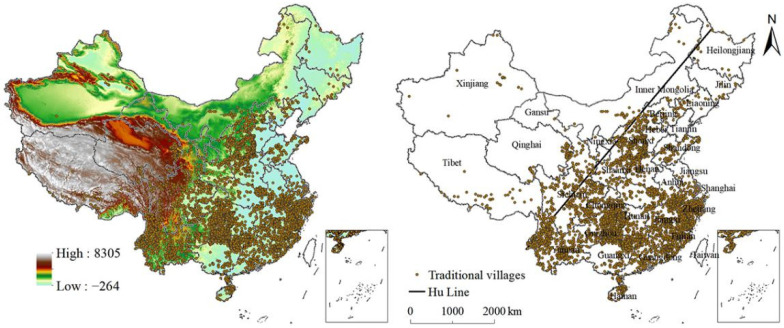
Spatial distribution of traditional villages in China.

The data of traditional villages were obtained from the Global Change Scientific Research Data Publishing System (http://www.geodoi.ac.cn/WebCn/Default.aspx, accessed on 9 April 2022) (Figure 1). Villages that have formed early, possess abundant traditional resources and complete traditional architectural features, maintain traditional characteristics in site selection and layout, have certain historical, cultural, scientific, artistic, social, and economic values, and have distinctive regional and traditional cultural characteristics can enter the directory of traditional villages in China. Based on this concept, the Ministry of Housing and Urban-Rural Development, Ministry of Culture, and Ministry of Finance announced five batches of traditional villages in China: 646 in 2012; 915 in 2013; 994 in 2014; 1598 in 2016; and 2666 in 2019 for protecting and developing these villages. Digital Elevation Model (DEM) was sourced from the National Earth System Science Data Sharing Platform (http://www.geodata.cn, accessed on 9 April 2022). Data on climate zones were obtained from the Earth Science Data Sharing Institute of Geographic Sciences and Natural Resources at the Chinese Academy of Sciences (http://www.resdc.cn/data.aspx?DATAID=264, accessed on 9 April 2022). Socioeconomic, humanistic, and historical data were obtained from the China Statistical Yearbook in 2017 published by the National Bureau of Statistics (http://www.stats.gov.cn/tjsj/ndsj/2017/indexch.htm, accessed on 9 April 2022). Data on the ethnic minority population were obtained from the sixth census of the National Bureau of Statistics (http://www.stats.gov.cn/tjsj/pcsj/rkpc/6rp/indexch.htm, accessed on 9 April 2022).

### 2.2. Methods

#### 2.2.1. Nearest Neighbour Index

To analyse the type of spatial distribution of traditional villages (e.g., random distribution, uniform distribution, or agglomeration distribution), this study used the nearest neighbour index to characterise the proximity of traditional villages in a geographic space [29]. The nearest neighbour index (*R*) is defined as the ratio of the average nearest neighbour distance to the theoretical nearest neighbour distance. *R* can be calculated as follows:

(1) R=r1rE

(2) rE=12nA

where *n* represents the number of traditional villages; *A* is the area of the study; *r*_1_ represents the average nearest neighbour distance, and *r_E_* represents the theoretical nearest neighbour distance. If *R* = 1, the point features are randomly distributed; if *R* > 1, the point features are uniformly distributed; and if *R* < 1, the point features are clustered.

#### 2.2.2. Geographic Concentration Index

The geographic concentration index (*G*) is generally used to indicate the degree of spatial distribution and agglomeration of objects [24]. The range of *G* lies between 0 and 100. The larger the value, the more concentrated the distribution of traditional villages. *G* can be calculated as follows:

(3) $$G=100\times \sqrt{\sum_{i=1}^{n}{\left(\frac{Y_{i}}{T}\right)}^{2}}$$

where *G* is the geographic concentration index of traditional villages, *Y_i_* is the number of traditional villages in the *i*-th provincial area, *T* is the total number of traditional villages, and *n* is the total number of provinces.

#### 2.2.3. Imbalance Index

The imbalance index (*S*) reflects the imbalanced distribution of traditional villages within each province [30]. It ranges from 0 to 1. *S* = 0 means that traditional villages are evenly distributed in each province; *S* =1 means that traditional villages are concentrated in one province. *S* can be calculated as follows:

(4) $$S=\frac{\sum_{i=1}^{m}Y_{i}-50(m+1)}{100m-50(m+1)}$$

where *Y_i_* is the cumulative percentage of the first *i* units in the descending order of the ratio of the number of traditional villages in each province to their total number and *m* is the total number of provinces.

#### 2.2.4. Kernel Density Analysis

This study used kernel density analysis to explore the spatial distribution density of traditional villages [31]. Kernel density analysis explores the distribution characteristics of samples by examining the regional spatial changes of sample point density. The results can identify the concentration and dispersion of the regional samples. Traditional villages within the search area were assigned different weights; those closer to the search centre were given greater weights. Kernel density can be calculated as follows:

(5) $$f_{h}(x)=\frac{1}{nh}\sum_{i=1}^{n}k(\frac{x-x_{i}}{h})$$

where *f_h_ (x)* is the kernel function, *h* > 0 is the bandwidth, and (*x* − *x_i_*) represents the distance of the estimated value x to the observed value *x_i_*. The kernel function calculates the aggregation of point elements in the entire area based on input data, reflecting the degree of influence of a kernel on the surroundings. This study used the kernel density tool integrated with the ArcGIS 10.8 toolbox for mapping.

#### 2.2.5. Pearson’s Correlation Analysis

To further verify the relationship between the influencing factors and the spatial distribution of traditional villages, this study conducted a Pearson’s correlation analysis and checked its statistical significance by using SPSS Statistics software [32]. The Pearson’s correlation coefficient can be calculated as follows:

(6) $$\rho_{x,y}=\frac{N\sum XY-\sum X\sum Y}{\sqrt{N\sum X^{2}-{\left(\sum X\right)}^{2}}\sqrt{N\sum Y^{2}-{\left(\sum Y\right)}^{2}}},$$

(7) $$t=\rho \sqrt{\frac{n-2}{1-\rho^{2}}}$$

where *ρ_x_*_,*y*_ is the Pearson’s correlation coefficient, *X* and *Y* are two variables, and *t* is the statistical significance. Variables with a correlation coefficient between −1 and 1 are said to be uncorrelated if they are close to 0, while they are strongly correlated if they are close to 1 or −1.

## 3. Results

### 3.1. Spatial Distribution Characteristics of Traditional Villages in China

Traditional villages vary greatly across provinces. The top five provinces with the most traditional villages were Guizhou, Yunnan, Hunan, Zhejiang, and Shanxi, accounting for 48% of the total number of traditional villages (Table 1). Tianjin, Ningxia, Shanghai, Jilin, and Heilongjiang provinces had the least number of traditional villages. In addition, the proportions of five batches in eastern China were 30.65%, 16.72%, 24.65%, 33.48%, and 28.17%, respectively. In central China, the proportions of five batches were 28.17%, 24.48%, 25.05%, 29.79%, and 47.11%, respectively, and 41.18%, 58.80%, 50.30%, 36.73%, and 24.72%, respectively, in western China. The nearest neighbour indices of the five batches of traditional villages were 0.402, 0.319, 0.388, 0.384, and 0.349, respectively. This indicates that traditional villages in China showed a trend of aggregated distribution in space. The geographic concentration indices of the five batches of traditional villages were 25.61%, 36.66%, 29.94%, 26.96%, and 27.76%, respectively. If traditional villages were evenly distributed in every province of China, the geographic concentration index would be 17.96%. It can be seen that the distribution of traditional villages was relatively concentrated from a national perspective.

**Figure 2:**
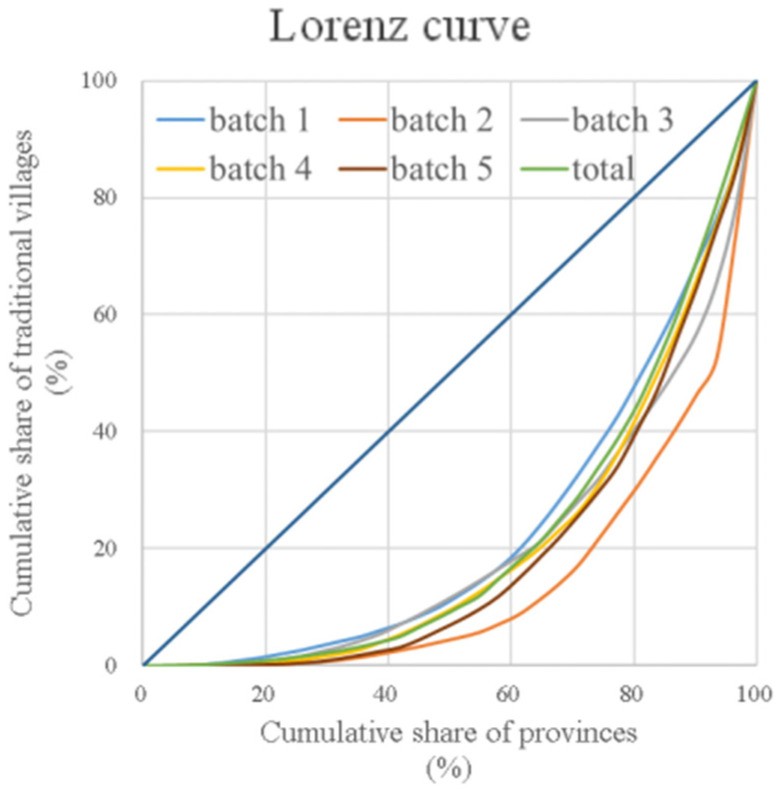
Lorenz curves of traditional villages.

According to Equation (4), the imbalance indices of the five batches of traditional villages were 0.42, 0.39, 0.41, 0.53, and 0.56, respectively; the imbalance index of all traditional villages was 0.59. This further confirmed that traditional villages were unevenly distributed on a national scale. The Lorenz curve was plotted using the cumulative percentage of traditional villages. The curve reflects the balance of the spatial distribution of traditional villages. As shown in Figure 2, the Lorenz curves of the five batches of traditional villages presented a typical concave form, indicating their unbalanced spatial distribution. The curve of the second batch of traditional villages had the highest degree of curvature and imbalance, indicating that the second batch of traditional villages had the most unbalanced spatial distribution with a higher concentration.

**Figure 3:**
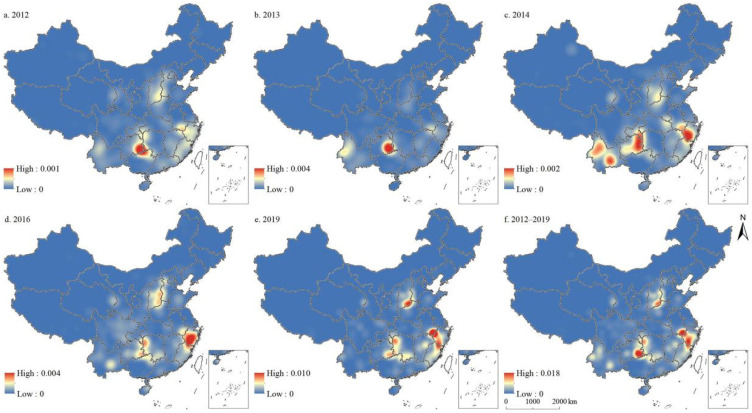
Distribution of kernel density of traditional villages in China.

Figure 3 presented the spatial distribution of the kernel density of the five batches of traditional villages in China. Overall, the kernel density of the five batches of traditional villages in China from 2012 to 2019 showed that most traditional villages were located in southeastern China and only a few were located in northwestern China. The distribution of traditional villages initially formed at the junction of Hebei, Shandong, and Henan provinces; the border between the Guizhou, Guangxi, and Hunan provinces; the border between the Anhui, Zhejiang, and Jiangxi provinces, and northwestern and southeastern Yunnan provinces.

Specifically, the kernel density results of the first batch of traditional villages formed two significant density cores: one at the junction of Hebei, Shandong, and Henan, and one at the junction of Guizhou, Guangxi, and Hunan. Two secondary cores formed at northwestern Yunnan and the junction of Anhui, Zhejiang, and Jiangxi. The kernel density in the southeast was greater than that in the northwest. The kernel density result of the second batch of traditional villages strengthened the core at the junction of Guizhou, Guangxi, and Hunan, while it was found to be weak in northwestern Yunnan. For the third batch of traditional villages, the kernel density analysis showed that the core at the junction of Guizhou, Guangxi, and Hunan existed to the east of Guizhou, while the core of Anhui, Zhejiang, and Jiangxi existed to the northwest of Zhejiang. Yunnan gradually formed two density cores in its northwestern and southeastern regions. The strength of the core at the junction of Hebei, Shandong, and Henan increased but was slightly weaker than that of the other cores. At the junction of Qinghai and Gansu, a secondary core was formed. The fourth batch of traditional villages weakened the core strength of northwest and southeast Yunnan so that the density core was biased towards southeast Yunnan. The core strength of Hebei, Shandong, and Henan increased significantly. The core of Anhui, Zhejiang, and Jiangxi moved eastward towards northwestern Zhejiang. The results of the fifth batch of traditional villages strengthened the core of Hebei, Shandong, and Henan so that it moved from southeast Shanxi to the junction of Shanxi and Henan. In addition, the core of Anhui, Zhejiang, and Jiangxi extended from northwest Zhejiang to Anhui.

### 3.2. Influencing Factors of Traditional Villages in China

#### 3.2.1. Socioeconomic Influencing Factors

##### Level of Economic Development

**Figure 4:**
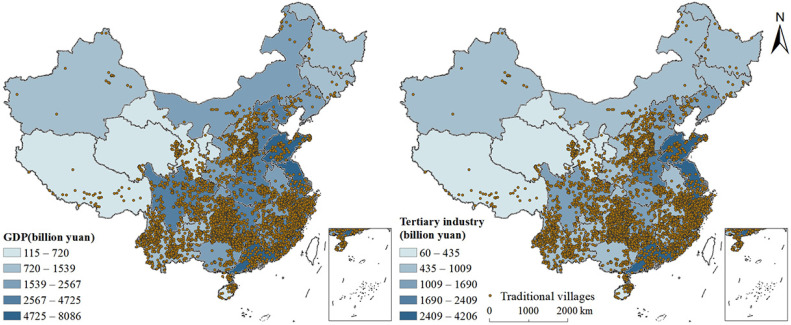
Spatial relationships between the distribution of traditional villages and economy.

Figure 4 showed that the Gross Domestic Product (GDP) and the output value of the tertiary industry show a decreasing trend from southeast to northwest China, which is consistent with the overall spatial distribution of traditional villages. Most traditional villages were concentrated in the economically developed areas of China. For example, the core at the junction of Anhui, Zhejiang, and Jiangxi belonged to the economically developed areas that retained many traditional villages. In areas with a high level of socioeconomic development, it was easier to spread the ideology of protecting historical and cultural heritage, which affected a better financial foundation to protect traditional villages from destruction. In contrast, there were a large number of traditional villages in some regions where the GDP and output value of the tertiary industry was relatively low, such as Yunnan province. This may be because of slow urbanisation, which promotes the retention of traditional villages. Therefore, regional economic growth and preservation of traditional villages are not contradictory in some regions.

To further verify the relationship between the level of economic development and the spatial distribution of traditional villages, this study conducted a Pearson’s correlation analysis. The Pearson’s correlation coefficient between the GDP and the number of traditional villages was 0.097, while the coefficient between the tertiary industry value and the number of traditional villages was 0.038; neither showed a significant association. Even though the analysis showed that the distribution of traditional villages was positively correlated with the economic development level, the impact of the regional economy on the distribution of traditional villages needs further analysis.

##### Transport Accessibility Conditions

**Figure 5:**
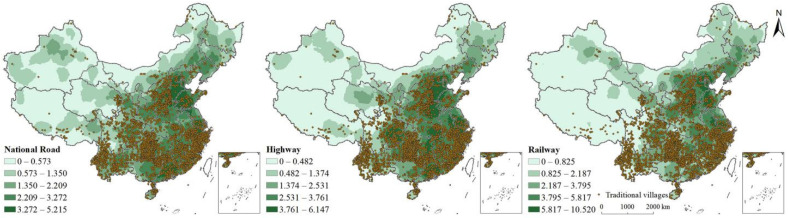
Spatial relationships between the distribution of traditional villages and locations.

We used the line density tool integrated with the ArcGIS spatial analyst to estimate the line density of the regional road network. Thereafter, the network densities of China’s railways, highways, and major roads were graded using the natural breaks method that was superimposed on the traditional villages (Figure 5). Two major cluster types in terms of the distribution of traditional villages and transportation conditions in China can be seen. First, some traditional villages were concentrated in the areas of China that were not closely connected to the outside world; the transportation conditions were generally underdeveloped and the location was inconvenient. However, these transportation conditions simultaneously retained the lifestyle of local residents and protected traditional buildings from being destroyed by development. In particular, some remote areas, such as Tibet, have complex terrain and climatic conditions that are not conducive to human habitation. Underdeveloped transportation conditions were not conducive to the preservation of traditional villages. Therefore, the number of traditional villages was positively correlated with transportation conditions in these areas. Second, some traditional villages were mainly concentrated in Hebei, Henan, Jiangsu, Zhejiang, Hubei, Sichuan, Chongqing, and other provinces that are represented by convenient transportation. Even though these traditional villages were in areas with convenient transportation and communication, they were well protected. However, the number of traditional villages in areas with well-developed transportation is lower than in areas with poor transportation. Overall, the number of traditional villages is negatively correlated with transportation in China (mainly in Sichuan, Zhejiang, and Anhui provinces).

The inheritance and protection of traditional villages were also inseparable from the infrastructural conditions in these regions. This study used the Pearson’s correlation analysis to obtain the correlation coefficients of six infrastructure indicators: the actual road length at the end of the year, the actual road area at the end of the year, urban bridges, urban drainage pipeline length, urban sewage treatment capacity, and urban road lighting. These were analysed along with the number of traditional villages. The correlation coefficients were −0.009, −0.013, 0.06, 0.028, −0.005, and 0.051, respectively. The correlation coefficients between indicators related to the road and the number of traditional villages were negative, which further proved that the spatial distribution of traditional villages was negatively related to transportation conditions, from a national perspective.

##### Population Size

**Figure 6:**
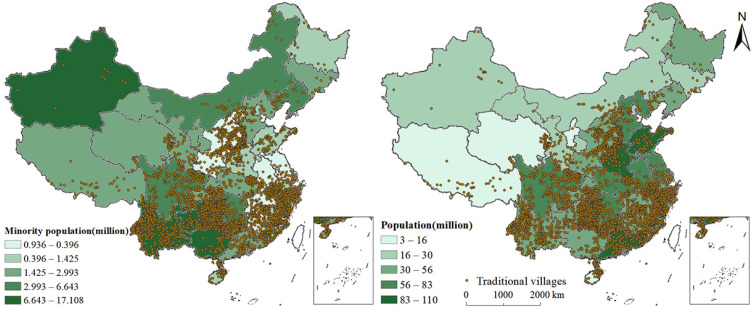
Spatial relationships between the distribution of traditional villages and population.

A Pearson’s correlation analysis of the total population and ethnic minority populations of each province and the number of traditional villages found that the coefficient between the total population and the number of traditional villages was 0.555, which was significant at a 0.01 level. The correlation coefficient between the number of ethnic minority populations and the number of traditional villages was 0.339, significant at a 0.05 level. Overlaying the thematic map of the national population with the distribution map of traditional villages (Figure 6) showed that more than 80% of the traditional villages were located east of the Hu line, which conformed to the distribution of population in China. In addition, the distribution of Chinese ethnic minorities was also roughly consistent with the distribution of traditional villages. They were mainly distributed in Guangxi, Yunnan, Xinjiang, and Guizhou. Yunnan and Guizhou were the areas with the highest numbers of traditional villages.

##### Distance from Central Cities

**Figure 7:**
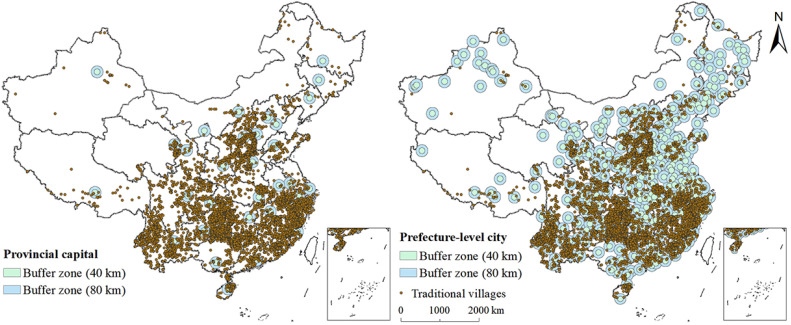
Spatial relationships between the distribution of traditional villages and cities.

This study selected 31 capital cities and 346 major cities as the central cities of China. The buffer tool in ArcGIS 10.8 was used to analyse the spatial relationship between traditional villages and their nearest central cities. As shown in Figure 7, the buffer radius established by each capital and major city was 40 km and 80 km, respectively. There were only 144 and 656 traditional villages within a radius of 40 km and 80 km from the capital cities, respectively, confirming that most of the villages were far from capital cities and were less affected by urban radiation. However, there were 2611 and 5344 traditional villages within a radius of 40 km and 80 km from major cities, respectively, accounting for approximately 78% of the total number of traditional villages. Therefore, it can be said that traditional villages were located at the urban boundary. This may be because it was conducive to the preservation of traditional villages, as transportation in the border area was not convenient. This spatial phenomenon showed that, to a certain extent, urban development was not conducive to the preservation of traditional villages. However, with the development of cities, the value, excavation, development, and protection of traditional villages in China have gradually received increased attention in recent years.

#### 3.2.2. Cultural and Historical Influencing Factors

##### Number of Cultural and Related Industrial Legal Entities above Designated Size

**Figure 8:**
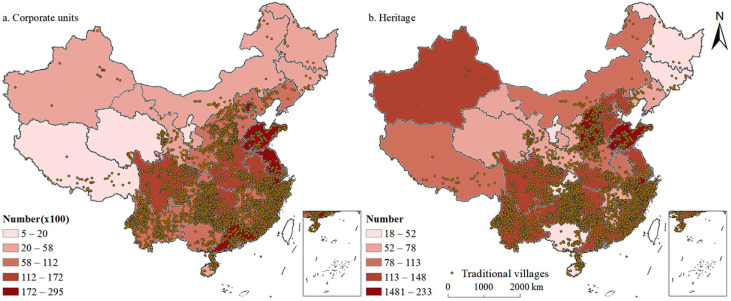
Spatial relationships between the distribution of traditional villages and humanities history of China.

This study used tools integrated with the ArcGIS spatial analyst to create a national-scale map that showed the number of cultural and related industrial legal entities (e.g., news and information services, content creation and production, or cultural entertainment and leisure services) above the designated size stipulated by the National Bureau of Statistics. We used the natural breaks method for grading and superimposed them on the traditional villages (Figure 8a). It can be seen that traditional villages were concentrated in areas where the number of cultural and related industrial legal entities above the designated size was moderately high. The Pearson’s correlation coefficient between the number of cultural and related industries above the designated size and the number of traditional villages was 0.399, significant at a 0.05 level and indicating that the number of cultural legal entities was an important factor affecting the spatial pattern of traditional villages in China. In particular, the number of cultural and related industrial legal entities over the designated size in coastal provinces was relatively high which was consistent with the spatial distribution of traditional villages.

##### Intangible Cultural Heritage

China has the largest number of intangible cultural heritage items in the world, with 3145 items of intangible cultural heritage, of which 656 belong to folk customs and folk literature, as defined by the Convention for the Safeguarding of the Intangible Cultural Heritage. Zhejiang (233 items), Shandong (173 items), Shanxi (168 items), Hebei (148 items), Guangdong (147 items), Jiangsu (145 items), and Guizhou (140 items) are the top seven provinces with the most intangible heritage items. Our results showed that provinces enriched with intangible cultural heritage had more traditional villages, which was consistent with the nuclear density analysis results of traditional villages in China. The Pearson’s correlation coefficient of the number of intangible cultural heritage items and the number of traditional villages in each province was 0.595, which was at a 0.01 significance level. This indicates that intangible cultural heritage was an important factor affecting the layout of traditional villages.

#### 3.2.3. Physical Influencing Factors

##### Altitude

**Figure 9:**
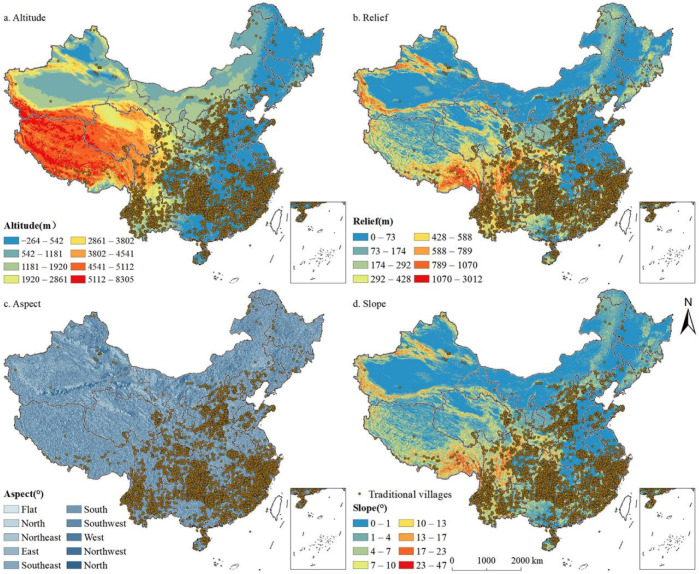
Spatial relationships between the distribution of traditional villages and natural geographical factors.

**Figure 10:**
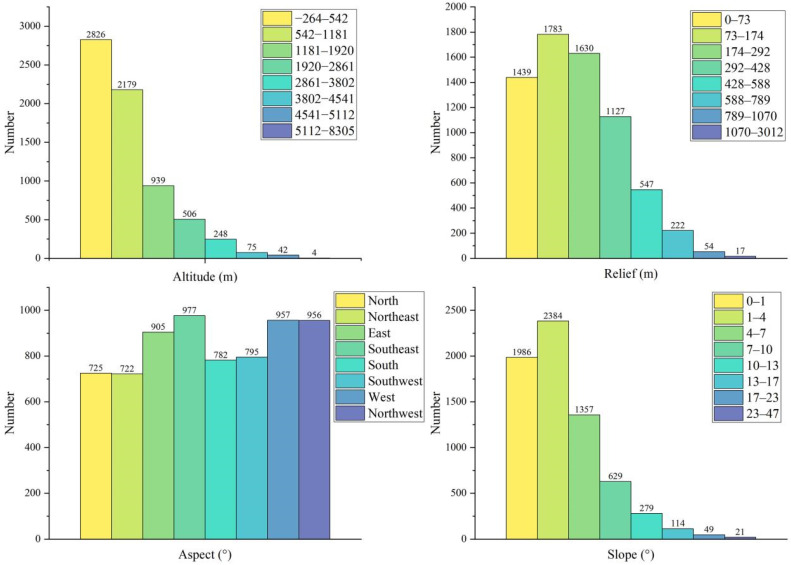
Statistics of traditional villages in different geographical conditions.

Altitude is an important attribute of traditional villages and an important index for quantitative research. Using ArcGIS 10.8 surface analysis slope tools, we reclassified China’s DEM data using natural breaks methods and superimposed it on traditional villages (Figure 9a). The map layout showed that generally, traditional villages were situated in low and medium altitude areas. By counting the number of traditional villages at different altitudes (Figure 10), we discovered that 2826 traditional villages were distributed in the interval with an altitude of less than 542 m, accounting for approximately 41% of the traditional villages; moreover, 73% of the traditional villages were located in areas below 1181 m altitude. The results showed that the higher the altitude, the fewer the number of traditional villages, indicating that the distribution of traditional villages is negatively correlated with altitude.

There are two types of traditional villages in China categorised in terms of altitude. One is high-altitude clustered villages, represented by traditional villages in the Yunnan and Qinghai-Tibet Plateau. For example, Yunnan is a high-altitude area with an average altitude of approximately 2000 m and retains many traditional villages. This type of traditional village is better preserved, largely because of the inconvenience of transportation in high-altitude areas, slow progress in industrialisation and urbanisation, and an underdeveloped economy that isolates traditional villages from the external environment. The other type consists of an agglomeration of villages represented by traditional villages in the plain areas of China. These traditional villages are generally located in the second and third steps of the Chinese terrain. The results showed that low-altitude areas represented by the central and eastern regions of China were more suitable for population settlement and village development.

##### Relief

This study used national DEM data and the ArcGIS 10.8 spatial analysis tool/neighbourhood analysis to extract the maximum and minimum altitudes in the analysis window. It then used the raster calculator tool to calculate their difference to generate a topographic relief map (Figure 9b). This map showed that traditional villages in China were concentrated in low-value areas with topographic undulations, as well as a gradual decrease in number from low level to a high level. Relief is an important indicator for human settlement adaptability evaluation that not only affects the location of traditional villages but also restricts population distribution, road transportation, economic development, and cultural exchanges, with profound effects on the spatial distribution of traditional villages.

Counting the number of traditional villages with various reliefs (Figure 10), it can be seen that there were 3222 traditional villages where the topographic undulations were between 0 and 174 m, indicating that traditional villages were densely distributed in regions with small topographic undulations and were sparse in regions with large topographic undulations. This may be due to the flat terrain, which is convenient for transportation and production conditions and has lower residential construction costs in areas with fewer undulations. These areas were extremely attractive for production and living activities that were conducive to the preservation of traditional villages.

##### Aspect

To address aspect, we used the ArcGIS 10.8 Surface Analysis/Aspect tool to analyse the Chinese DEM data. Reclassification of aspect extraction of the Chinese DEM data resulted in eight categories: north, northeast, east, southeast, south, southwest, west, and northwest (Figure 9c). We performed an overlay analysis to obtain the number of traditional villages on various slope areas (Figure 10). It can be seen that the choices of traditional villages are diverse. Traditional villages were evenly distributed in this aspect, and therefore, they do not have a positive orientation.

##### Slope

Using the slope analysis tool in ArcGIS, the Chinese DEM data were extracted for slope and reclassified by natural breaks methods, to be superimposed on the traditional village (Figure 9d). Overall, most traditional villages are located in low-slope areas. Observing the number of traditional villages with different slopes (Figure 10) between 0° and 10°, we found 6356 traditional villages, accounting for 93% of the total number of traditional villages. Between 10° and 17°, there were 393 traditional villages, accounting for 6% of the traditional villages. The number of traditional villages with slopes greater than 17° was only 70, accounting for 1% of the total number of traditional villages. Therefore, the layout of Chinese traditional villages tends to be situated on low-gradient slopes in China, especially in coastal plains where the production conditions and living environment can meet the developmental needs of traditional villages.

##### Climate

**Figure 11:**
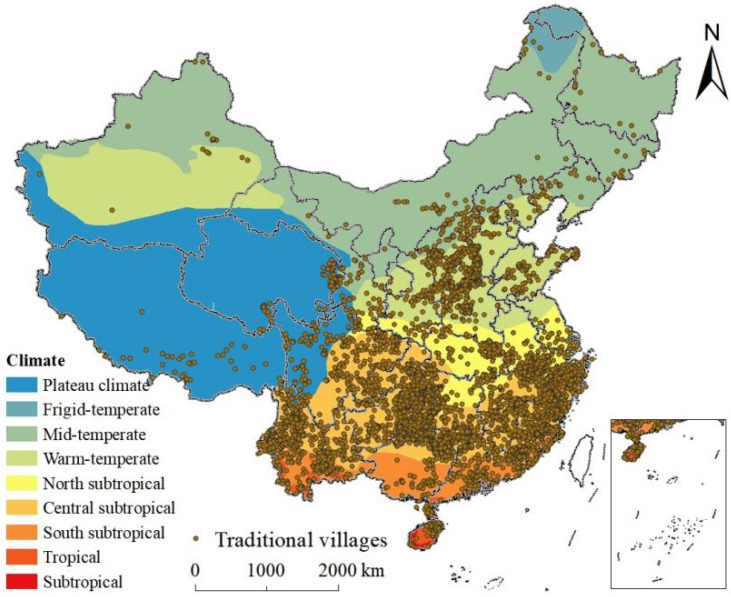
Spatial relationships between the distribution of traditional villages and climate.

Southern, eastern, central, and northern China located in the subtropical and warm temperate zones were the agglomeration areas of traditional villages (Figure 11). Areas with subtropical climate are located to the south of the Qinling Mountain-Huai River Line and to the east of the Qinghai-Tibet Plateau and are affected by the monsoon climate, rain, and heat, which is conducive to the large-scale development of production and living. Further, there is a warm temperate zone, affected by the Pacific monsoon, located to the north of the Qinling Mountains—Huai River Line and the middle and lower reaches of the Yellow River. Summers are hot and rainy, while winters are cold and dry. Therefore, the monsoon was conducive to the generation and preservation of traditional villages.

## 4. Discussion

### 4.1. Value of Protecting Traditional Villages

Traditional villages in China have rich material and intangible cultural heritage, unique historical and cultural values, scientific research and educational values, economic tourism values, and natural landscape resources. Protecting traditional villages serves to protect the country’s precious historical and natural resource heritage [33,34,35]. The value of traditional villages lies in the following aspects:

(1) Every traditional village is rich in culture and heritage, with non-renewable and potential tourism resources that reflect the cultural essence and spatial memory of harmonious coexistence between man and nature.

(2) In traditional villages, farmers can cultivate agricultural lands according to the local climate. They organically combine local soil, geology, and farming techniques to develop many unique traditional products with local flavours. For example, West Lake Longjing Tea is representative of China’s high-quality agricultural and side-line products carried by traditional villages.

(3) Traditional villages form the foundation of the development of rural tourism, thereby increasing employment opportunities and the income of local villagers and providing more financial protection. There are many successful cases of promoting local economic development through the development of rural tourism in China, in places such as Sichuan, Zhejiang, and Fujian.

(4) Traditional villages provide vivid samples for the study of archaeology, history, architecture, folklore, sociology, and local vernacular culture. They are ingeniously integrated with the surrounding natural landscape elements. Undoubtedly, such traditional villages are precious resources.

Traditional villages have geographic embeddedness of architectural form; that is, architectural form and structure are constrained by the local natural environment, as it tends to harmonise with the environment [36,37]. Traditional villages are still well preserved in relatively poor areas or places with inconvenient transportation. In an abstract sense, the preservation of traditional villages is inversely proportional to the modernisation of the countryside. With modernisation, the geographic embeddedness of village buildings has been gradually replaced by geographical de-embedding [36,38]. Due to improvements in the transportation infrastructure and a decrease in the cost of new building materials, people can go beyond the limitation of the local geographical environment to choose new building materials. In this situation, Wang (2017) believed that once the villagers acquire wealth, they often have the urge to replace traditional buildings with modern ones, which can cause a decline in traditional villages [36]. The villagers believe that modern architectural forms indicate prosperity, so the decline in traditional villages is related to not only the trend of modernisation and marketisation but also the villagers’ ideas. While some people consider the disappearance of traditional village buildings to be a tragedy because it leads to a loss of historical and cultural values. The protection of traditional villages depends on the power relationship between the two groups and their compromise to achieve an intermediate solution. Most people who seek protection for traditional villages live in cities, while most rural residents are eager to seek modernisation. Therefore, a key to the protection of traditional villages is to find a joint force between the two groups in China.

### 4.2. How to Protect and Develop Traditional Villages in China

The results showed that traditional villages present different morphological characteristics in space that is influenced by geographical environment factors and human factors. Different protection priorities and paths should be established for different villages. For example, intangible cultural heritage items are important influencing factors in the evolution of traditional villages. For traditional villages with rich intangible cultural heritage items, we should increase their historical and cultural publicity, and pay attention to the construction of roads, so as to vigorously develop tourism. While for traditional villages with rich intangible cultural heritage items that have become tourist attractions, we should pay more attention to protecting the original features of villages. Considering the role of environmental factors, those traditional villages closer to the city should be prevented from being over-commercialized and losing their character. While for traditional villages in remote locations, such as those located in Tibet, Guizhou, and other regions with poor transport facilities, we should increase investment in policy funds to improve people’s livelihood and improve the village’s self-development ability by bringing in the talents to protect the traditional villages.

Traditional villages in China face multiple challenges, including accelerating urbanisation, urban-rural integration, and the modernisation of rural construction. The government should establish a protection system and protection department for traditional villages as an important policy for the integration of urban and rural areas and the construction of villages [39]. All functional departments should perform their duties and cooperate to protect, utilise, and manage traditional villages, change the multi-departmental management system of traditional villages, and establish a government-based and enterprise-assisted social participation protection mechanism. It should be strictly forbidden to demolish and merge traditional villages or destroy any protected objects, and a strict permission system for rural construction planning must be implemented. Any projects in traditional villages must meet the requirements of protection and development planning in China [40]. Priority should be given to the protection of endangered cultural relics, historical buildings, and other aspects related to cultural heritage in traditional villages. Therefore, we also need to change the traditional concepts of the villagers and make them realize that it is beneficial to them to preserve the original features of the villages.

As a concept and method of protecting large-scale cultural landscape heritage [41], “heritage area”, can be considered established in the integration of traditional villages that are geographically adjacent. Based on the discussion of the spatial distribution of traditional villages, we can divide a wider range of heritage areas. Within the protected areas, buildings that seriously affect the overall appearance may be demolished, to allow for new buildings that are in harmony with the original buildings in appearance. The appearance of uncoordinated buildings outside core protected areas can be appropriately modified and should not be demolished on a large scale [42]. We highly recommend strengthening the improvement of traditional villages’ environment. It is essential to improve the traditional village patterns and ecological environment to strengthen infrastructure construction and improve the production and living conditions of villagers. However, we must meet the control requirements of traditional village styles. Tourism and leisure are important ways to protect and utilise traditional villages; however, these activities must be executed in a moderate and well-organised manner. All localities should manage the relationship between resource carrying capacity, villagers’ acceptance, economic tolerance, and the protection of the villages’ cultural heritage appropriately to oppose the excessive commercialisation of the villages. Furthermore, diversified, socialised, and transferable protection models should be developed to actively explore and implement diversified protection methods, such as village self-protection, private protection, and public assistance. The effective protection of traditional villages must be combined with environmental improvement, the development of tourism, and cultural industries. It is necessary to protect the cultural and natural heritage of traditional villages effectively and implement development and utilisation projects to change the poverty and underdevelopment of traditional villages.

### 4.3. Validations and Limitations of the Study

Previous studies on the spatial pattern of traditional villages using GIS analysis and mathematical models have achieved significant results. However, compared with other branches of geography, rural settlement science has received less attention due to insufficient research efforts. There is a lack of analyses of the spatial distribution pattern of traditional villages and their influencing factors on a national scale from a geographical perspective. Therefore, this study used the GIS and spatial analysis to examine the spatial distribution characteristics and influencing factors of traditional villages in China on a national scale, thereby providing theoretical support for their protection and development. However, this study did not discuss the external form, internal structure, and architectural features of traditional villages, or the emotional connection of the villagers with these villages. In addition, the construction of beautiful villages, characteristic towns, and the new national urbanisation strategy have been proposed, providing new opportunities and development ideas for rural development. Under the context of new urbanisation, further research on characteristic resources of traditional villages is of great significance to the construction of urban and rural planning and rural geography.

## 5. Conclusions

To meet the need for the protection and development of traditional villages, this study explored the distribution pattern of all five batches of traditional villages based on the Geographic Information System and spatial analysis with mathematical statistics. We also further discussed the influence of socioeconomic factors, cultural and historical factors, and physical factors on the distribution of traditional villages, to thereby provide support for their protection and development.

Our findings reveal that there is a clear spatial difference between traditional villages. Specifically, the number of traditional villages in the northeastern and western regions of China—including Sichuan, Shaanxi, Ningxia, Gansu, Qinghai, Tibet, and Xinjiang—was relatively small. Then, the results of Pearson’s correlation analysis showed that GDP and the tertiary industry value were not closely related to the number of traditional villages, though the distribution of traditional villages was positively correlated with the economic development level. The spatial distribution of traditional villages was negatively related to transportation conditions from a national perspective, but significantly positively correlated with the total population, ethnic minority populations, number of cultural and related industrial legal entities above designated size, and intangible cultural heritage of each province. Special attention should be paid to the correlation coefficient between the number of intangible cultural heritage and the number of traditional villages in each province was the highest, indicating that intangible cultural heritage is an important factor affecting the layout of traditional villages. We also found that most of the traditional villages were far from capital cities but close to major cities, and distributed in the geographical conditions of low altitude, low relief, and low slope. Traditional villages form an inseparable whole with the natural, cultural, economic, and social environment in which they live. In a word, the spatial agglomeration and pattern formation of traditional villages in China come from the result of the long-term interaction of these factors. This study effectively explored the spatial distribution characteristics and influence mechanism of traditional villages, which can be the basis for policy-making for traditional villages’ sustainable revitalization.

## Figures and Tables

**Table 1:** Number of traditional villages announced in different batches in China.

Provinces	Batch 1	Batch 2	Batch 3	Batch 4	Batch 5	Total
Tianjin	1	0	0	2	1	4
Ningxia	4	0	0	1	0	5
Shanghai	5	0	0	0	0	5
Jilin	0	2	4	3	2	11
Heilongjiang	2	1	2	1	8	14
Xinjiang	4	3	8	2	1	18
Beijing	9	4	3	5	1	22
Liaoning	0	0	8	9	13	30
Jiangsu	3	13	10	2	5	33
Tibet	5	1	5	8	16	35
Inner Mongolia	3	5	16	20	2	46
Gansu	7	6	2	21	18	54
Hainan	7	0	12	28	17	64
Shaanxi	5	8	17	41	43	114
Chongqing	14	2	47	11	36	110
Qinghai	13	7	21	38	44	123
Shandong	10	6	21	38	50	125
Henan	16	46	37	25	81	205
Hebei	32	7	18	88	61	206
Hubei	28	15	46	29	88	206
Guangdong	40	51	35	34	103	263
Guangxi	39	30	20	72	119	280
Sichuan	20	42	22	141	108	333
Jiangxi	33	56	36	50	168	343
Anhui	25	40	46	52	237	400
Fujian	48	25	52	104	265	494
Shanxi	48	22	59	150	271	550
Zhejiang	43	47	86	225	235	636
Hunan	30	42	19	166	401	658
Yunnan	62	232	208	113	93	708
Guizhou	90	202	134	119	179	724
In total	646	915	994	1598	2666	6819

## Data Availability

Not applicable.
